# Development of a Digital Content-Free Speech Analysis Tool for the Measurement of Mental Health and Follow-Up for Mental Disorders: Protocol for a Case-Control Study

**DOI:** 10.2196/13852

**Published:** 2020-05-14

**Authors:** Peter Tonn, Yoav Degani, Shani Hershko, Amit Klein, Lea Seule, Nina Schulze

**Affiliations:** 1 Neuropsychiatric Center of Hamburg-Altona Hamburg Germany; 2 VoiceSense Ltd Herzelia Israel

**Keywords:** voice detection, depressive disorder, content-free speech analysis, mobile health app

## Abstract

**Background:**

The prevalence of mental disorders worldwide is very high. The guideline-oriented care of patients depends on early diagnosis and regular and valid evaluation of their treatment to be able to quickly intervene should the patient’s mental health deteriorate. To ensure effective treatment, the level of experience of the physician or therapist is of importance, both in the initial diagnosis and in the treatment of mental illnesses. Nevertheless, experienced physicians and psychotherapists are not available in enough numbers everywhere, especially in rural areas or in less developed countries. Human speech can reveal a speaker’s mental state by altering its noncontent aspects (speech melody, intonations, speech rate, etc). This is noticeable in both the clinic and everyday life by having prior knowledge of the normal speech patterns of the affected person, and with enough time spent listening to the patient. However, this time and experience are often unavailable, leaving unused opportunities to capture linguistic, noncontent information. To improve the care of patients with mental disorders, we have developed a concept for assessing their most important mental parameters through a noncontent analysis of their active speech. Using speech analysis for the assessment and tracking of mental health patients opens up the possibility of remote, automatic, and ongoing evaluation when used with patients’ smartphones, as part of the current trends toward the increasing use of digital and mobile health tools.

**Objective:**

The primary objective of this study is to evaluate measurements of participants' mental state by comparing the analysis of noncontent speech parameters to the results of several psychological questionnaires (Symptom Checklist-90 [SCL-90], the Patient Health Questionnaire [PHQ], and the Big 5 Test).

**Methods:**

In this paper, we described a case-controlled study (with a case group and one control group). The participants will be recruited in an outpatient neuropsychiatric treatment center. Inclusion criteria are a neurological or psychiatric diagnosis made by a specialist, no terminal or life-threatening illnesses, and fluent use of the German language. Exclusion criteria include psychosis, dementia, speech or language disorders in neurological diseases, addiction history, a suicide attempt recently or in the last 12 months, or insufficient language skills. The measuring instrument will be the VoiceSense digital voice analysis tool, which enables the analysis of 200 specific speech parameters, and the assessment of findings using psychometric instruments and questionnaires (SCL-90, PHQ, Big 5 Test).

**Results:**

The study is ongoing as of September 2019, but we have enrolled 254 participants. There have been 161 measurements completed at timepoint 1, and a total of 62 participants have completed every psychological and speech analysis measurement.

**Conclusions:**

It appears that the tone and modulation of speech are as important, if not more so, than the content, and should not be underestimated. This is particularly evident in the interpretation of the psychological findings thus far acquired. Therefore, the application of a software analysis tool could increase the accuracy of finding assessments and improve patient care.

**Trial Registration:**

ClinicalTrials.gov NCT03700008; https://clinicaltrials.gov/ct2/show/NCT03700008

**International Registered Report Identifier (IRRID):**

PRR1-10.2196/13852

## Introduction

### Human Language

Human language is a fundamental ability which, in addition to social interaction, primarily serves the exchange of feelings; however, in the sense of an inner dialogue, it also serves as a means of self-perception and self-reflection [1]. This establishes a relationship between pure content as an expression of cognitive performance and emotional content in the sense of affective meaning. This close connection between emotion and cognition, especially with language and speech, has been scientifically researched within the framework of relational frame theory for many years [2]. Human language as a form of expression, with human listening on the other side, has been the basis of professional therapeutic support for mental disorders for more than 100 years [3,4]. The recent treatment developments regarding linguistic and psychological research in relational frame theory have been acceptance-based treatments, such as acceptance and commitment therapy [5]. 

Due to the increasing global frequency of mental disorders, their safe detection and treatment is very important. According to World Health Organization calculations [6], depression alone will be the second greatest burden of disease in the world in 2020. Other mental disorders, such as neurodevelopmental disorders (attention deficit hyperactivity disorder [ADHD], autism spectrum disorders, etc [7,8]) or personality disorders [9], which have been increasing in recent years, also cause significant individual stress and have a societal impact due to the loss of potential productivity and treatment costs [10]. Early detection and treatment of mental disorders is, therefore, a major factor in preventing these problems, as is the avoidance of deterioration. However, there is currently no objective, ideal physiological parameter available for this task.

Since the 1990s, attempts have been made to determine the emotional state of a speaker by analyzing the content of their speech and the linguistic structure [11]. There have been positive results thus far, with high specificity and sensitivity in some subjects when using suitable parameters, like in cases where depressive disorders have been detected [12-14]. As a result, there has been intensive development in the automation of such analyses, with continuing positive results [14]. However, so far dissemination of such analyses, such as in the early detection, diagnosis, or assessment of mental disorders, has not occurred in broad clinical practice but has been described in a few single cases [15,16]. The reasons for this may be complex, but it may be due to the high technical effort required to perform these analyses that have led to them not being well reflected in outpatient care practice [17]. Also, resentment against machine diagnosis or very technical measurements may have played a role that should not be underestimated. Finally, the diagnostic and prognostic validity of such measures still needs to be proven.

In the last decade, electronic assistants have taken up considerable space in all areas of life, first through computerization and later via smartphones [18]. In medicine, and especially in psychiatry and psychotherapy, numerous programs, online tools, and coaching applications are now available and have seen increasing acceptance [19]. At the same time, the technical effort that needs to be expended to create a high-quality, medically or psychologically useful application for use in the clinic has decreased [20,21]. The applications that can be implemented in this way are even more complex and extensive than previously thought possible. It may, therefore, make sense to use these new technical possibilities and examine whether the analysis of language, especially the noncontent aspects (eg, speech flow, speech melody, expression), can be used by a differentiated algorithm to assess the mental state of a subject. It is possible that they could also measure the course of mental illness, the quality of life of patients with chronic mental illnesses, and patients' well-being [22].

The study presented here uses a noncontent linguistic analysis algorithm developed by VoiceSense, a company specializing in prosodic speech analysis, which links speech patterns to behavioral tendencies. The analysis is commercially used for personal risk assessment by banks and insurance companies, for candidate assessment by human resources companies, and customer analytics by enterprise call centers. The current study would provide a structured setting for the differentiated evaluation of psychopathological factors (effects, personality aspects, psychomotor factors, etc) by this speech pattern analysis.

### Research Objectives

The primary objective of this study is to evaluate the measurements of the mental state of the participants by comparing the analysis of noncontent speech parameters to the results of several psychological questionnaires (Symptom Checklist-90 [SCL-90], the Patient Health Questionnaire [PHQ], and the Big 5 Test). We hypothesized that the emotional state of mind, the behavioral patterns, the well-being, and some of the personality traits of the participants could be identified using the prosodic analysis algorithm. A second hypothesis is that the diagnosis could be confirmed by the application of the analysis program, and a distinction can be made between different psychopathologically-defined syndromes.

## Methods

### Participants

A total of 166 outpatients will be recruited in one center (Neuropsychiatric Center of Hamburg), following the invitation of their treating physician or psychotherapist, to use a new method to measure their mental state and distress. Inclusion and exclusion criteria for the study are listed in Textbox 1.

**Inclusion and exclusion criteria.**

Inclusion criteria:

- A regular outpatient with a mental disorder or neurological disease
- Native German speaker
- Aged 18-65 years old
- In good general health (absence of cancer, acute myocardial infarction, unstable angina, severe cardiac arrhythmia, recent cerebrovascular incident, or severe atherosclerosis).

Exclusion criteria:

- Schizophrenia
- Dementia
- Current or recent (less than 1 year) history of alcohol or drug abuse
- Current or recent (less than 1 year) history of suicide attempts
- Other significant comorbidities according to the Investigator’s clinical assessment (eg, cancer, acute myocardial infarction, unstable angina, severe cardiac arrhythmia, recent cerebrovascular incident, or severe atherosclerosis).

For the exclusion criteria, both schizophrenia and dementia have shown indications that a patient’s speech patterns are influenced by these disorders. Therefore, we decided to consider these two groups of patients separately in a future study to see whether there is a comparable difference to the general population on a content-free linguistic level.

### Linguistic Algorithm and Recording System

#### Audio Collection

Speech utterances of the subjects will be sampled using the VoiceSense mobile app, which will be installed on the tablets (BENEVE Co, 10.1-inch screen, 32 gigabytes of memory, Android version 7.0) of the research examiners. The recordings will be taken over 1-3 separate sessions throughout the study: the initial interview, the first follow-up session after 2-4 weeks, and the second follow-up session after another 2-4 week period. In each of those sessions, the research examiners will activate the app by logging into it with a dedicated username and password that was prepared in advance for each subject (the username and password do not reveal the subject’s identification). The app then presents 9 general questions (eg, “Please describe in a few sentences how was your day yesterday”). The subject presses record and answers, and when finished they will press stop and the app will present a second question for the subject to answer. The app will count the recorded time to verify that at least 120 seconds are recorded, but if after nine answers there is still not enough recording time then the app will present an increasing number of questions till the time requirement is met. Once enough speech is recorded, the app will send the recorded audio to the VoiceSense cloud server for analysis. The content of the subject’s answers is not important for the analysis; therefore, the questions are designed in a general manner to enable collection of the natural speech patterns of the subjects. The recordings will be done in a quiet room in the Neuropsychiatric Center Hamburg, which is reserved for diagnostic purposes. The examiner will leave the room while the recording is done, to make the subjects feel more comfortable and to not influence their free speech.

#### Speech Analysis

The audio that will be sent to the server will be analyzed using VoiceSense proprietary speech analysis. This analysis produces raw data of over 200 prosodic parameters for each call. The raw data parameters will be sent back to the research center for the statistical analysis, with a sampling frequency of 8 kilohertz.

VoiceSense speech analysis focuses on speech prosody, the noncontent aspects of the speech, such as intonation, pace, stressing, and other aspects. The analysis is language independent and has been tested successfully in many languages (different European languages, different Asian languages, etc). The analysis was validated versus various personality tools, such as Big 5 [23], Holland [24], Hogan [25], WPI [26], and linked speech patterns (clusters of the raw speech parameters) with the personality scales as measured by these tools. The analysis is based on several granted patents regarding measurement of emotional state [27] and behavioral tendencies [28] through speech analysis. This proprietary speech anal ysis is the basis for commercial products in the fields of enterprise, big data analytics, customer analytics, human resources, and others.

### Study Design and Data Protection

This study is a case-controlled study with a case group and a control group to identify the best target population for measuring speech patterns with the VoiceSense app. We adhered to the Consolidated Standards of Reporting Trials (CONSORT) guidelines in the design of the trial.

In the first step, the participants will be grouped by the physician or psychotherapist into a control group (participants without mental disorders) or a case group (participants with mental disorders). All participants will provide written informed consent after being given detailed information by the treating therapist or physician. The original forms will be stored in a closed cabinet, but the participants will receive a copy. Second, they will receive a pseudonymized label for the blinded inhouse-rater to work with the questionnaires, and for the data center for the speech-analysis. Neither the rater nor the data center will know the name and diagnosis of the participant. Data security and availability will be ensured every time, according to the European Union (EU) rules [29]. The participants’ data will be stored in a MySQL-Database. Within the project database, identifying data will be stored separately from the collected data, with only project staff with specifically conferred access rights able to access the identifying data. It is envisioned that the distribution of participants will correspond to the clinical distribution in the Neuropsychiatric Center with different degrees of severity of mental disorders (about 25% mild, about 50% moderate, about 25% severe). This should be checked by the study nurse every 2 weeks.

For documentation purposes, the presence or absence (in the case of neurological outpatients) of any psychiatric diagnosis will be screened using the PHQ-D [30]. Personality traits will then be measured with the Big 5 Test [23]. In the case of ADHD, we added a Visual Analogue Scale (ADHD-VAS) to identify the three main affective states (impulsivity, inattention, and hyperactivity). This will be done at baseline only.

**Figure 1:**
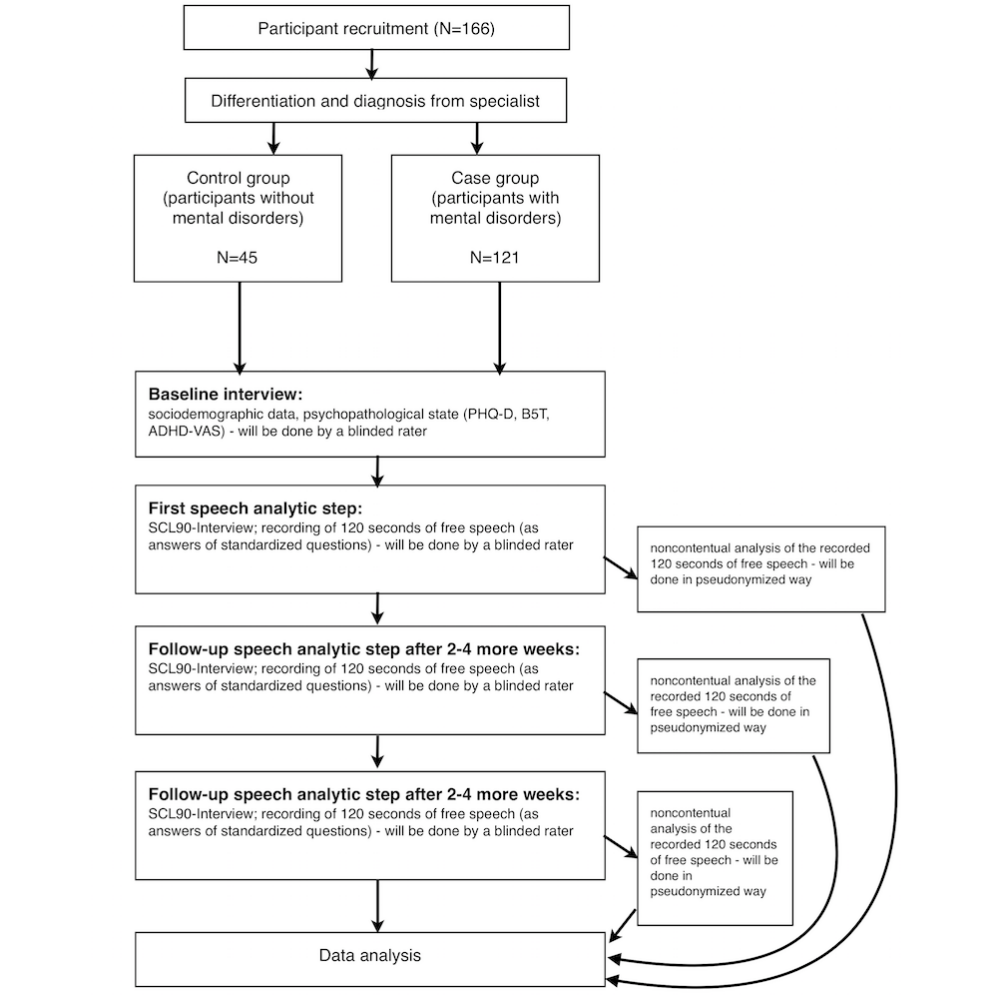
Flowchart of the study protocol. PHQ-D: Patient Health Questionnaire. B5T: Big 5 Test. ADHD-AS: attention deficit hyperactivity disorder-visual analogue scale. SCL-90: Symptom Checklist-90.

At baseline, at follow-up one (2-4 weeks after baseline), and follow-up two (2-4 weeks after follow-up 1), participants will be measured for mental distress using the SCL-90 [31]. They will then be asked to freely answer nine standardized questions, with a few sentences per question. These answers will be recorded for evaluation. If less than 120 seconds of free speech is possible, three more standardized questions will be asked and the answers to these will also be recorded. The recorded sentences will then be sent to the server to carry out the prosodic analysis. The results of the questionnaires and the results of the prosodic analysis will be stored in the patients’ records on file. Figure 1 shows the flow chart of the study.

### Statistical Methods

It should be possible for the study team to evaluate the efficacy and effectiveness of the raw data from the linguistic analysis in correlation with the clinical data collected with the questionnaires. All statistical tests will be two-tailed and will be considered statistically significant at *P*<.05. A sample size of 166 subjects was calculated (effect size medium=0.30; alpha=0.05; 1–beta=0.95), and considering a drop-out rate of 15%, a total sample size of 190 patients was determined. 

Statistical analysis will be performed with R version 3.5.3 (The R Project, Vienna, Austria), and epidemiological data will be evaluated. Continuous variables will be described with the mean, standard deviation, median, minimum, maximum, and the 25th and 75th percentiles. Categorical variables will be described with percentages and absolute frequencies. The differences in continuous variables between the two groups will be evaluated with the Kruskal-Wallis test, followed by the Dunn multiple comparison test. The differences between the two diagnostic groups (psychiatric versus neurological diagnosis) for normally distributed data will be evaluated with a one-way analysis of variance (ANOVA), followed by the Newman-Keuls multiple comparison test. The normality of the distribution will be evaluated with the Kolmogorov-Smirnov test. Any correlation between the variables under evaluation will be assessed by the Spearman r correlation. To compare qualitative data, we will use the Chi-square test with the Yates correction or the Fisher exact test. Lastly, the variables will be grouped to their diagnostic groups according to the 10th edition of the International Statistical Classification of Diseases and Related Health Problems or the fifth edition of the Diagnostic and Statistical Manual of Mental Disorders, and a confirmative factor analysis will be done comparing these groups with the prosodic parameters.

### Ethics Approval

This study protocol has been approved by the ethical committee of the Neuropsychiatric Center under the Declaration of Helsinki. Written informed consent will be collected from the participants before they will be included in the study.

## Results

The study is ongoing as of September 2019, and as of this date, we have enrolled 254 participants. The dropout rate has been acceptable, with 161 participants having thus far completed every questionnaire and the speech analytical measurements at time point one. Overall, 67 participants have reached time point two and 62 participants have reached the end of the study. We believe we can close recruitment and start final statistical calculations by November 2019.

## Discussion

The prevalence of mental disorders has increased in recent decades, and thus their importance in society has also grown. This can be traced back to the reduced stigma surrounding them and the associated growing acceptance, as well as to a growing social awareness of the problem. Undoubtedly, early detection in the development of mental illness, as well as adequate follow-up for relapse prevention, is one of the most important tools in reducing the burden of disease for the individual as well as for society. If the use of linguistic analysis, in the presented form of an app or as an online tool, can enable the possibility of validating the emotional state and behavioral patterns of a patient, this screening could be used for an initial assessment. Also, due to its low threshold of access, it could end up providing valuable support for those who might not otherwise be able to access any assessment.

The importance of linguistic parameters for the detection of the emotional or behavioral state of a patient has been proven in the light of previous literature, at least for patients with depressive disorders. However, the analyses carried out with content-free voice recordings have thus far been associated with a high level of technical and personnel expenditure. This study wants to examine whether mental distress measured by voice analysis compared with standard questionnaires could be an appropriate tool with validated results, and also whether other mental disorders have specific linguistic patterns (in addition to patients with depression) which could differ from each other.

## Abbreviations

ADHD: attention deficit hyperactivity disorder
ANOVA: analysis of variance
CONSORT: Consolidated Standards of Reporting Trials
EU: European Union
PHQ: Patient Health Questionnaire
SCL-90: Symptom Checklist-90
VAS: visual analog scale
